# BIOLOGICAL AGE INFLUENCES HEART FAILURE PATHOGENESIS

**DOI:** 10.1093/geroni/igz038.3246

**Published:** 2019-11-08

**Authors:** Kathleen Woulfe, Jacob Zumo, Ross Cook, Benjamin McNair, Jacob Schlatter, Emily Schmitt, Vikram Chhatre, Danielle Bruns

**Affiliations:** 1 University of Colorado-Denver, Aurora, Colorado, United States; 2 University of Washington School of Medicine, WWAMI Regional Medical Education, Seattle, Washington, United States; 3 University of Wyoming, Laramie, Wyoming, United States

## Abstract

Heart failure (HF) impacts patients of all ages and is an enormous public health problem. Historically, HF has been treated with a single, multi-purpose approach, despite the observation that biological differences such as age influence HF pathogenesis and therapeutic outcomes. We hypothesized that HF pathogenesis differs across the life-course, a hypothesis which we tested with a mouse model of cardiac dysfunction at three distinct stages of life. C57BL/6 mice at pediatric (5 weeks), adult (3-5 months), and old (18 months) ages were treated with a mini-osmotic pump that eluted isoproterenol (ISO; 30mg/kg/hour) for six days. As expected, cardiovascular morbidity and mortality were significantly worse in the old group. Both pediatric and adult underwent hypertrophic remodeling, as evident by higher LV weight relative to tibia length (TL). However, ISO exposure did not increase LV/TL in old mice. We performed RNA-sequencing to understand pathways and genes differentially regulated by age. 119, 1515, and 33 genes were significantly differentially expressed in pediatric, adult, and old mice exposed to ISO, respectively. Of these, only 2 transcripts were upregulated in response to ISO across all three ages. Expression of pro-fibrotic mediators differed across the life-course, with adults inducing a pro-fibrotic transcriptional program (α-smooth muscle actin, fibronectin, collagen, periostin) that was attenuated in old and absent in pediatric animals. Our data clearly demonstrates that pediatric, adult, and aged hearts activate distinct molecular remodeling in response to ISO, highlighting the significance of age as a biological variable in HF pathogenesis.

